# An experimental test of the importance of isolated trees for frog diversity in tropical landscapes

**DOI:** 10.1111/1365-2656.70081

**Published:** 2025-06-12

**Authors:** Mauricio Almeida‐Gomes, Andreza Soares de Siqueira, Vitor Nelson Teixeira Borges‐Júnior, Maria Anita Tozzi, Rodrigo da Fonseca da Silva, Marcus Vinícius Vieira, Jayme Augusto Prevedello

**Affiliations:** ^1^ Instituto de Biociências Universidade Federal de Mato Grosso do Sul Campo Grande Brazil; ^2^ Departamento de Ecologia Universidade do Estado do Rio de Janeiro Rio de Janeiro Brazil; ^3^ Laboratório de Vertebrados, Departamento de Ecologia Universidade Federal do Rio de Janeiro Rio de Janeiro Brazil; ^4^ Knowledge Center for Biodiversity Belo Horizonte Minas Gerais Brazil

**Keywords:** Anura, artificial ponds, community structure, habitat fragmentation, matrix quality, scattered trees

## Abstract

1. Isolated trees are conspicuous in fragmented landscapes and are often considered ‘keystone structures’, due to their significant ecological role despite occupying small areas. They are also regarded as ‘biodiversity foci’, because they may support higher abundance and species richness than nearby open areas. However, these suggestions have not been tested experimentally yet. Using experimental ponds and amphibians as a model system, we tested the importance of isolated trees for biological communities in a biodiversity hotspot, the Atlantic Forest.
2. We built and sampled 28 artificial ponds, 10 near the edge of a continuous forest, nine beneath isolated trees and nine in open pasture. To test for differences in amphibian community structure among experimental treatments, we quantified community abundance, local species richness and community composition. We also tested whether the abundance of the four numerically dominant species in the ponds (*Physalaemus signifer*, *Leptodactylus latrans*, *Rhinella ornata* and *Scinax* aff. *x‐signatus*) differed among treatments. Finally, we compared total (landscape‐level) species richness among treatments.
3. Ponds located beneath isolated trees and near forest edges had a higher community abundance and abundance of *P. signifer* and *R. ornata*, as well as a higher total (landscape‐level) species richness than ponds located in open pasture. Ponds beneath isolated trees had a similar community composition to ponds near the edge of the continuous forest, whereas open pasture communities had a markedly different community composition.
4. Our results provide experimental evidence that isolated trees contribute to increase total (landscape‐level) species richness and community abundance of anurans in ponds in deforested areas, making local communities more similar to those found near forest edges.
5. We recommend protecting and planting isolated trees across deforested areas, particularly near natural ponds, as well as restoring ponds near isolated trees. These relatively low‐cost actions can contribute substantially to increasing the abundance and species richness of anuran communities in fragmented landscapes.

## Graphical abstract

We performed the first experimental test of the importance of isolated trees for biodiversity. Our results provide evidence that isolated trees contribute to increasing total species richness and community abundance of anurans in artificial ponds, making local communities more similar to those found in ponds near forest edges.

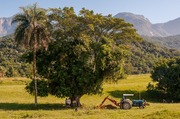

## 1 INTRODUCTION

Land use and land cover changes (LULCC) have profoundly altered the structure of landscapes worldwide, causing widespread habitat loss and fragmentation, with impacts on biodiversity and the provision of ecosystem services (Di Pirro et al., 2021; Shah et al., 2023). Such LULCC have been intensified in recent decades due to the growing demand for goods and services (Mansour et al., 2020), leading to the conversion of natural habitats into modified habitats, including matrix areas that are less suitable for native species, such as pastures, crops and urban areas (Assede et al., 2023). By 2100, LULCC are likely to be primary sources of negative impacts on biodiversity in the tropics, overcoming the effects of climate change (Sala et al., 2000). Thus, actions that reduce or mitigate LULCC, particularly habitat loss and fragmentation, are urgently needed (Lindenmayer et al., 2008; Shipley et al., 2023).

A potentially simple and relatively low‐cost action proposed recently to mitigate habitat loss and fragmentation is conserving and planting isolated trees (Manning et al., 2006; Prevedello et al., 2018). Isolated trees, also known as scattered, remnant or pasture trees, occur in a wide range of landscapes around the world, both in natural (e.g. savannas) and human‐dominated areas, such as pastures (De‐Carvalho et al., 2021; Manning et al., 2006; Prevedello et al., 2018). In human‐modified landscapes, isolated trees may represent the few remnants of previously forested areas that were cleared for farming (Fischer et al., 2010). Isolated trees provide local‐scale ecosystem services, such as regulating microclimate, acting as nuclei of forest restoration and providing a variety of resources for animals (Manning et al., 2006). In addition, isolated trees contribute to increasing forest cover at the landscape‐level and may increase landscape connectivity by functioning as stepping stones between forest patches (Cadavid‐Florez et al., 2020; Manning et al., 2006). Therefore, isolated trees can help maintain animal communities in modified landscapes.

For these reasons, isolated trees have been regarded as ‘keystone structures’ in fragmented landscapes, as they have a large ecological importance despite the small areas they occupy (Manning et al., 2006). For example, matrix areas with isolated trees may support species richness, abundance and community composition similar to that of sampling areas within habitat patches (Prevedello et al., 2018). Moreover, isolated trees have been suggested as ‘biodiversity foci’, as they usually support higher species abundance and richness than nearby open areas in both forest and non‐forest biomes (Dunn, 2000; Manning et al., 2006; Prevedello et al., 2018). However, despite the increased recognition of the importance of isolated trees for biodiversity (Prevedello et al., 2018), no study has experimentally tested the hypotheses that isolated trees function as keystone structures or biodiversity foci. Experimental tests are important to identify the mechanisms underlying the higher richness and abundance usually found near isolated trees (Prevedello et al., 2018), since such tests allow controlling for potentially confounding factors. Experimental tests are also urgently needed as isolated trees are threatened by illegal logging in human‐modified landscapes (Manning et al., 2006). Understanding the impact and relevance of the presence of these trees in maintaining biodiversity may be essential for conservation, especially in highly degraded and species‐rich regions, such as the global biodiversity hotspots (Myers et al., 2000).

Here, we report the results of the first experimental test of the importance of isolated trees for biodiversity in human‐modified landscapes. To do so, we experimentally created ponds in matrix areas and tested how the presence of isolated trees near these ponds affects animal communities, using amphibians as model organisms. Amphibians may be interesting models for testing the impact of landscape composition, including the presence of isolated trees, on animal communities, for several reasons. Amphibians are the group of terrestrial vertebrates with the highest proportion of threatened species (Luedtke et al., 2023), with habitat loss and fragmentation among the largest threats to their populations (Cushman, 2006). In addition, amphibians can respond to different environmental conditions, such as pond size, hydroperiod, forest cover and the presence of isolated trees (e.g. Almeida‐Gomes et al., 2016; Semlitsch et al., 2015). Amphibians have higher rates of evaporative water loss (due to their permeable skin) and lower dispersal ability relative to other vertebrates (Rothermel & Semlitsch, 2002). Usually, open habitats have higher temperatures and lower soil moisture compared to forest areas (Li et al., 2015), hampering the occurrence of some amphibian species. In fact, a previous study found that most forest‐dependent frogs were not found in open pasture (Almeida‐Gomes & Rocha, 2014). In addition, LULCC may reduce the availability of reproductive sites for amphibians, affecting several species that depend on specific habitats for reproduction that are only found in forest areas (Almeida‐Gomes & Rocha, 2015).

In this scenario, the presence of trees near ponds may be especially important for the occurrence of some frog species, for several reasons. First, trees near ponds can provide suitable microclimatic conditions and microhabitats, which can positively affect the survival and reproduction of frogs (Gould et al., 2024). Second, the leaves that fall from the trees can represent an important resource subsidy to frog biomass (Earl & Semlitsch, 2012). Third, isolated trees can be used as stepping stones and short‐term refugia by some frogs (Chan‐McLeod & Moy, 2007). Indeed, a previous observational study indicated that the presence of trees near temporary ponds may increase the richness and abundance of anuran species (Almeida‐Gomes et al., 2016). Therefore, isolated trees may increase frog diversity in matrix areas in human‐modified landscapes.

To experimentally evaluate the importance of isolated trees in maintaining frog diversity in deforested areas, we built 30 artificial ponds in a fragmented landscape of the Atlantic Forest, a top‐ranked global biodiversity hotspot. The Atlantic Forest has more than 600 amphibian species, nearly 80% of them endemic to the biome (Haddad et al., 2013). Ponds were built in carefully selected pasture areas in three contrasting environments: near the edges of a continuous forest; beneath the canopy of isolated trees; and in open pasture. By comparing the abundance, species richness and composition of frog communities in these environments, we were able to experimentally test both the ‘keystone structures’ and the ‘biodiversity foci’ hypotheses. If the keystone structures hypothesis is true, we can expect similar species richness, abundance and composition between ponds located near the edge of the continuous forest and those beneath isolated trees, as isolated trees may provide microclimatic conditions and microhabitats similar to those found near forest edges. If the biodiversity foci hypothesis is true, we can expect higher species richness and abundance in ponds beneath isolated trees than in ponds in open areas, as well as a different species composition between these treatments, because open areas without trees may lack suitable microclimatic conditions and microhabitats for the occurrence of forest‐dependent species.

## 2 MATERIALS AND METHODS

### 2.1 Study site

**FIGURE 1:**
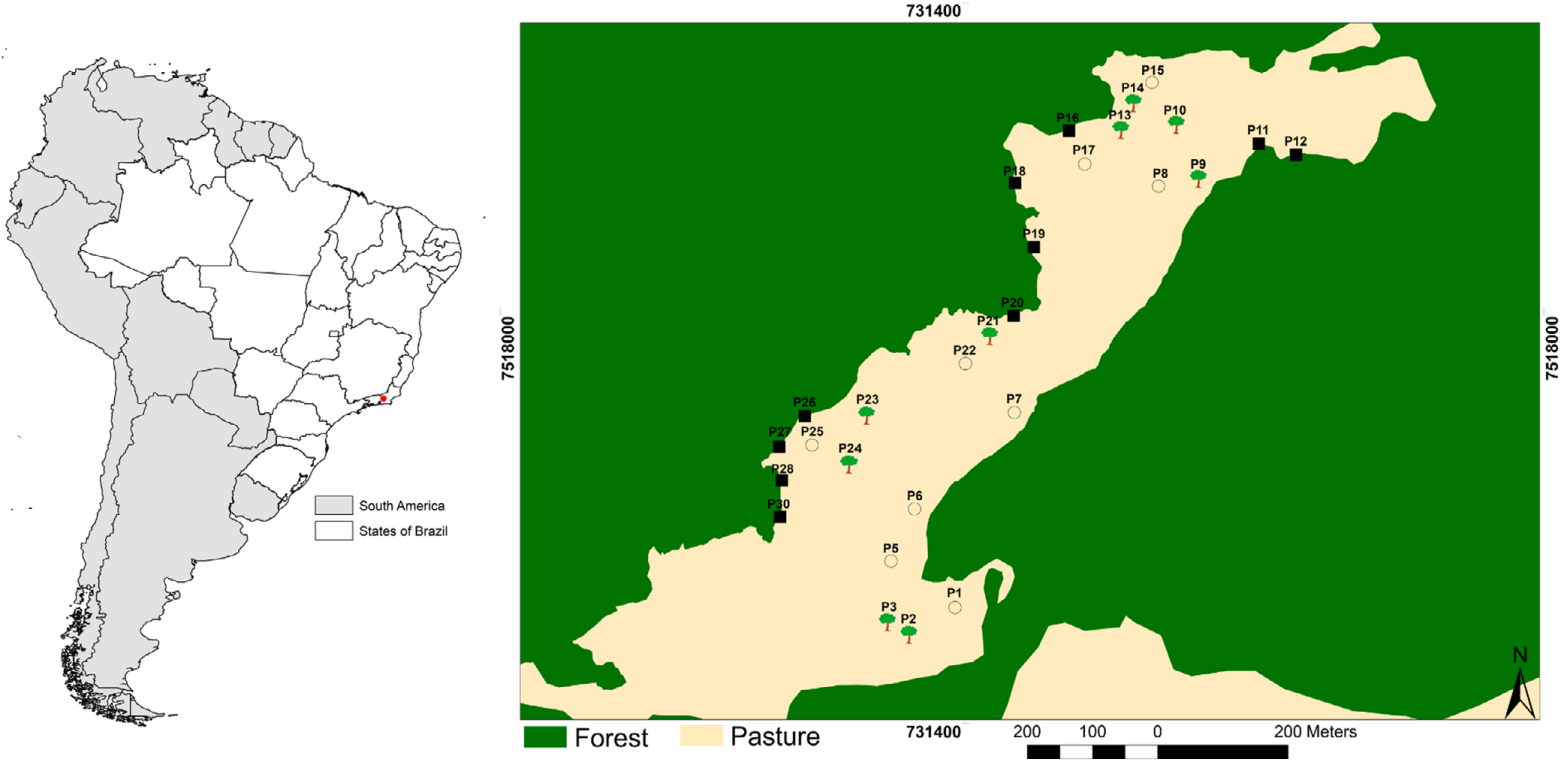
Study area showing the distribution of the 28 experimental ponds in an Atlantic Forest landscape in Cachoeiras de Macacu, Rio de Janeiro, Brazil (red dot). Ten ponds were located near the edge of the continuous forest (black squares), nine beneath isolated trees (tree symbols) and nine in open pasture (open circles).

The study area is located in the municipality of Cachoeiras de Macacu near the Reserva Ecológica de Guapiaçu (WGS 1984 UTM Zone 23S: 731381.86 m E; 7517910.01 m S), a private reserve located in the Atlantic Forest of Rio de Janeiro state, Brazil. The vegetation in the study area is classified as dense evergreen forest and the climate is mild‐humid‐mesotermic (Vieira et al., 2009). There are two distinct climatic seasons in the region, a relatively dry and cold season, from April to September and a wet and warm season, from October to March (Almeida‐Gomes & Rocha, 2014). The elevation in the study area ranges from approximately 50 to 80 m. The region has a large continuous forest area (~100,000 ha) and forest fragments of different sizes, immersed in different types of matrix, mainly pastures (Vieira et al., 2009). In these pastures, there is semi‐confined or unconfined cattle ranching (Vieira et al., 2009). The pasture area where the study was conducted is adjacent to the continuous forest (Figure 1) and includes several isolated trees of native species, predominantly *Ficus* spp. and *Guarea* spp., which are also common in the continuous forest areas.

### 2.2 Experimental design

**FIGURE 2:**
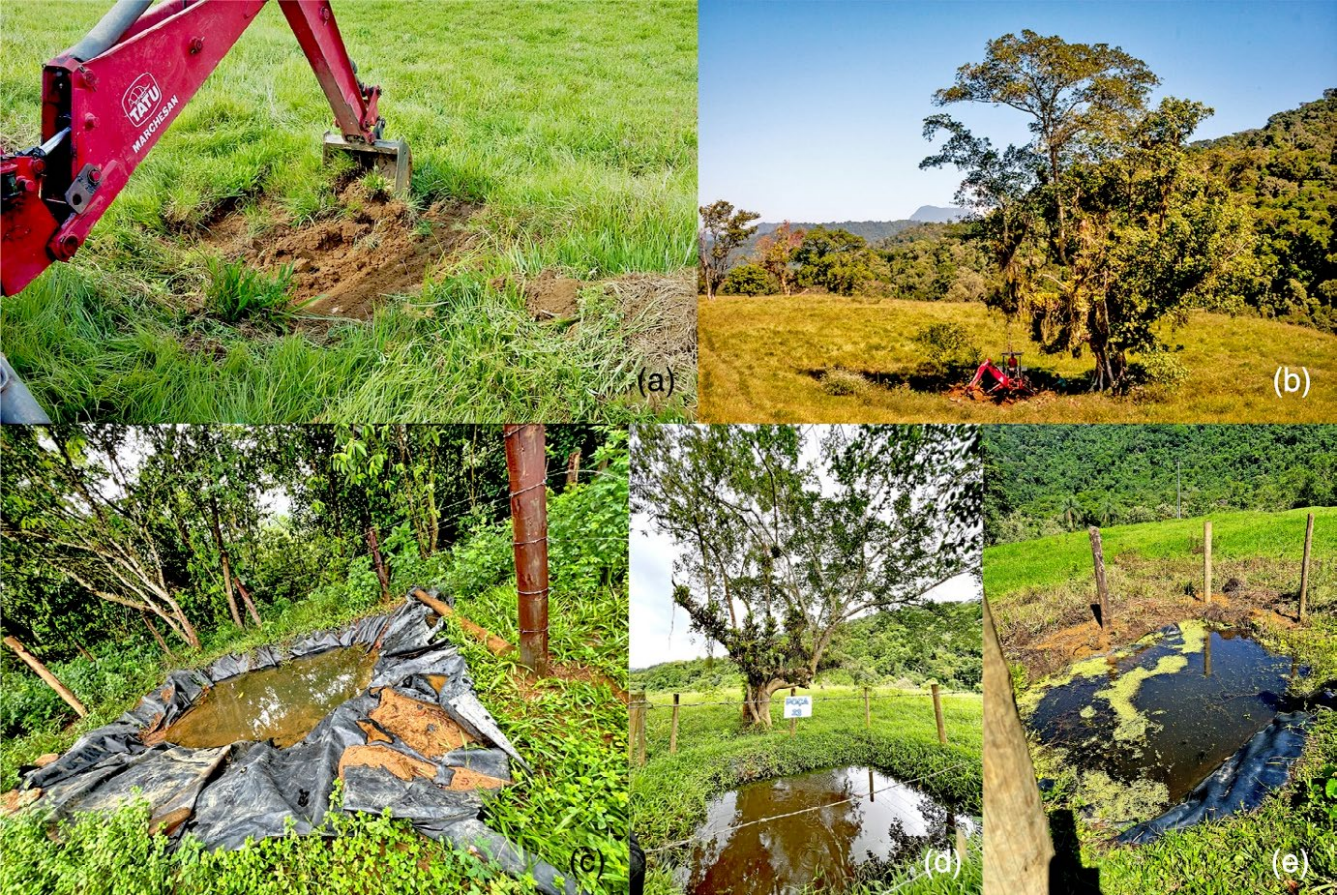
Illustration of the experimental system. The experimental ponds were built with a bulldozer (a, b) in three treatments: Near the edge of a continuous forest (c), beneath isolated trees (d) or in open pasture areas (e).

We built 30 artificial ponds in April 2016 with a bulldozer, all with approximately the same dimensions (about 3.0 m long × 2.5 m wide × 0.5 m deep), to control for possible influences of pond size on amphibian communities. This pond size (approximately 7.5 m^2^) falls within the range of experimental pond sizes used in previous studies on amphibians (Boone et al., 2004: 80 m^2^; Silva, Oliveira, et al., 2012: 1.5 m^2^; Rowland et al., 2017: nearly 1.81 m^2^). All ponds were lined with black plastic canvas and a layer of soil to allow rainwater to accumulate. Ten ponds were built near the edge of the continuous forest (up to 0.5 m from the edge; hereafter ‘edge ponds’), 10 beneath isolated trees (up to 2 m from a single *Ficus* tree; hereafter ‘tree ponds’) and 10 in open pasture areas (at least 20 m away from the nearest isolated tree; hereafter ‘open ponds’) (Figure 2). Most tree ponds were located near a single tree, but three ponds were built near a *Ficus* tree and up to two other non‐*Ficus* trees. To standardize the identity of focal isolated trees and reduce variability among tree ponds, only individuals from *Ficus* spp. were selected. Besides being abundant in the pasture matrix, these trees accumulate bromeliads that can provide varied resources and habitats for amphibians, such as vocalization sites and reproductive habitats (Bourne et al., 2001). Moreover, *Ficus* trees may be better agents for forest restoration than other remnant trees in human‐modified landscapes (Cottee‐Jones et al., 2016). The edge ponds had one side facing the forest and the other three sides facing the pasture area. Ponds were placed at the edge of the forest, rather than inside the forest, for two reasons. First, since a bulldozer was required to construct the ponds, it was logistically impossible to dig them inside the forest. Second, since we were interested in evaluating the importance of isolated trees for frog diversity, all ponds (including edge ponds) were surrounded by the same land cover (pasture areas). One experimental pond in open pasture (P29) and one beneath an isolated tree (P4) did not accumulate water and were excluded from the statistical analyses (Table S1). Distances between edge ponds (*N* = 10) ranged from 51.2 to 965.5 m (mean = 457.7 m), from 38.1 to 884.6 m (mean = 475.3 m) for tree ponds (*N* = 9) and from 87.7 to 859.8 m (mean = 419.4 m) for open ponds (*N* = 9). Considering all ponds together (*N* = 28), the distances ranged from 38.1 to 965.5 m (mean = 437.1 m).

To ensure that the experimental design was adequate, we first compared forest cover and distance to the continuous forest between tree and open ponds, since these variables can affect pond colonization by forest species. Edge ponds were not included in this test, as they obviously had higher forest cover (mean 45.7%) and lower distance to the edge of the forest (up to 0.5 m). The distance was calculated using *Google Earth Pro* 7.3.2, and forest cover around each pond was calculated using ArcGIS 10.6, using circular buffers of 100‐m radius (considering only patches above 0.08 ha or 800 m^2^). Forest cover ranged from 9.0% to 38.7% (mean 23.7%) for open ponds and from 0% to 29.7% (mean 14.7%) for tree ponds. Distance to the forest ranged from 20.6 to 75.5 m (mean 46.1 m) for open ponds and from 27.9 to 97.1 m (mean 56.8 m) for tree ponds. It was not possible to fully standardise the distance to the forest edge for tree and open ponds, as all ponds had to be constructed on flat terrain, which excluded some areas. Moreover, the distances of the tree ponds also varied due to the heterogeneous location of *Ficus* trees in the pasture area. Importantly, ANOVA tests indicated no difference between tree and open ponds in terms of both forest cover and distance to the continuous forest (Figures S1 and S2). In addition, there was no difference among the three treatments in terms of hydroperiod (i.e. number of sampling days in which the pond had accumulated water), average height of surrounding pasture vegetation, average pond edge slope and water depth (Figures S3–S6). The absence of differences among treatments for all these variables indicates that the experimental design was adequate and that any differences in amphibian communities may be attributed to the treatment effect, rather than other potentially confounding factors. Moreover, the results remained similar when these variables were included as covariates in the models (Figures S7 and S8; see Section 2.4).

### 2.3 Frog sampling and marking

Sampling was carried out between May 2017 and May 2018, totaling five sampling events and encompassing both dry and wet seasons. Adult anurans were sampled using the time‐limited visual encounter surveys method during the night (beginning at 7:00 p.m.). During the visual search, we inspected both the pond area and the terrestrial vegetation within 1.5 m of the pond edge. Trees were inspected to a height of 1.5 m, as frogs beyond this height could not be captured. Each sampling event consisted of three consecutive night surveys and was repeated every 3 months. For each pond, 5 min of active search were performed per day, totaling 75 min of effort per pond summing all five sampling events. To avoid double counting individuals at the same pond, each manually captured frog was marked on the left thigh with a fluorescent tag containing a unique alphanumeric code (Alpha Tags; developed by Northwest Marine Technology, Inc). These tags (1.2 mm × 2.7 mm) are implanted using the VI Alpha Injector and have been widely used in studies with fish (e.g. Niva, 1995; Summers et al., 2006) and frogs (Heard et al., 2008; Knapp et al., 2023; Vallejos et al., 2024), with no negative impacts reported on these animals. For example, females of the emerald glass frog can behave normally after tagging, even across multiple years (Vallejos et al., 2024). Moreover, there was 80% retention of VI Alpha tags over 1 year in larval Eastern Hellbenders (*Cryptobranchus alleganiensis alleganiensis*) (Knapp et al., 2023). All captured frogs were released after marking at the same location where they were found. Because we marked all individuals and our focus was not on reproduction, we did not sample juveniles or tadpoles. All capture and handling protocols were conducted in accordance with ICMBio/SISBIO (licence 13088‐1) and the study did not require ethical approval.

To evaluate sample completeness, we calculated sample coverage (the proportion of the total number of individuals that belong to the species detected in the sample) for each pond using the iNEXT package (Hsieh et al., 2016) and the exponent *q* = 0 for the Hill numbers (species richness). This analysis showed that our ponds were properly sampled, as the mean sample coverage for all ponds was 0.89 (range: 0.18–1.00). Only two ponds had sample coverage below 0.70 (P5 = 0.67 and P20 = 0.18). These two ponds were retained for analysis because their local species richness (P5 = two species; P20 = four species) fell within the range observed across the 28 sampled ponds (one to six species).

### 2.4 Data analysis

To statistically assess differences in community structure among experimental treatments, three complementary dependent variables were analysed: (i) local species richness (number of recorded species per pond), (ii) community abundance (number of recorded individuals across all species per pond) and (iii) community composition (detailed below). To better understand possible differences in community structure, we also performed additional analyses focused on the abundance per pond of four species in particular: *Leptodactylus latrans*, *Physalaemus signifer*, *Rhinella ornata* and *Scinax* aff. *x‐signatus*. These species were selected as they were the most abundant species in the experimental system and vary in terms of habitat preferences, with *P. signifer* and *R. ornata* preferring forest areas, whereas *L. latrans* and *S*. aff. *x‐signatus* were more common in open habitats (Almeida‐Gomes & Rocha, 2014; Haddad et al., 2013).

For the six dependent variables (local species richness, community abundance and the abundance of the four numerically dominant species), we built Generalized Linear Models (GLMs), with treatment as the explanatory factor, using likelihood‐ratio chi‐square tests for model comparison. We checked the occurrence of over‐ or underdispersion using the function check_overdispersion in the performance package (Lüdecke et al., 2021). For local species richness, we assumed Poisson distribution (with a log‐link function), because species richness was a count variable (Zuur et al., 2009) and the data had no over‐ or underdispersion. For the other five dependent variables, we built GLMs with negative binomial distribution and log‐link function, due to the presence of overdispersion. Assumptions of all GLMs were met, as inferred from analyses of model residuals (qqplots and plots of fitted vs. predicted values). Similar results were obtained when either local variables (hydroperiod and vegetation height; Figure S7) or landscape‐level variables (distance to the continuous forest and forest cover; Figure S8) were included as covariates of treatment in the models. These covariates can affect amphibians for different reasons. First, forest cover and hydroperiod can positively affect frog species richness and species composition, respectively (Almeida‐Gomes et al., 2016). Second, plants surrounding ponds can influence the distribution and abundance of amphibians (Burrow & Maerz, 2022), and most amphibians that use natural ponds in the study area have arboreal habits (Almeida‐Gomes et al., 2016). Finally, the distance to forest cover can influence pond colonization (Guerry & Hunter, 2002). For all dependent variables, post‐hoc pairwise differences between treatments were tested using the function contrast from the R package emmeans (Lenth, 2025) and Wald tests. Finally, we also compared total (landscape‐level) species richness among treatments, by counting the total number of species found in each treatment (a single value per treatment), considering all ponds of each treatment.

To visually assess differences in frog community composition among treatments, we used a principal coordinate analysis (PCoA), implemented in the R package ape (Paradis & Schliep, 2019). This method is considered more flexible than other multivariate analysis techniques for various types of data, as it can accommodate any distance or similarity measure. Moreover, it can identify potential groups or subgroups within the data based on similarity patterns, which was our primary goal. To compare statistically the composition between each pair of treatments, we performed a permutational analysis of variance (PERMANOVA), using the adonis function in the vegan package (Oksanen et al., 2024). In both cases, we used abundance data (rather than presence‐absence) and the Bray–Curtis distance. Therefore, differences in species composition among treatments could be caused by variation in species abundances and/or species identity. An ANOVA based on multivariate dispersions (calculated using the function betadisper in the vegan package) confirmed that variances were homogeneous among treatments (ANOVA: *F*_2,25_ = 0.28; *p* = 0.76). All statistical analyses were performed in R version 4.3.2 (R Core Team, 2024).

## 3 RESULTS

We recorded 273 frog individuals from 14 species belonging to three families in the 28 ponds (Tables S2 and S3). We recorded all species (*N* = 14) and 174 individuals in the wet season, and six species and 99 individuals in the dry season (Table S2). We recorded 38 recaptures for eight species (Table S4). The most recaptured species were *P. signifer* (*N* = 15) and *L. latrans* (*N* = 14) (Table S4). For *P. signifer*, we recorded one movement from a tree pond to an edge pond (163.9 m). For *L. latrans*, we recorded one movement from an edge pond to a tree pond (131.6 m) and one from an edge pond to an open pond (505.5 m; Table S4).

### 3.1 Species richness and abundance

We found 93 individuals of 11 species in tree ponds, 125 individuals of 10 species in edge ponds and 55 individuals of 6 species in open ponds. In tree ponds, community abundance ranged from 6 to 16 individuals (mean ± SD = 10.3 ± 4.0) and local species richness from one to six species (mean ± SD = 2.9 ± 1.8), with *Physalaemus signifer* (48.4% of the total number of individuals) and *Leptodactylus latrans* (23.6%) being the most abundant species in this treatment. In edge ponds, community abundance ranged from 4 to 24 individuals (12.5 ± 5.7) and local species richness ranged from two to five species (3.6 ± 1.1), with *P. signifer* (51.2%) and *Rhinella ornata* (17.6%) being the most abundant species. Finally, in open ponds, community abundance ranged from 2 to 11 individuals (6.1 ± 2.8) and local species richness ranged from two to three species (2.4 ± 0.5), and the most abundant species were *L. latrans* (52.7%) and *Scinax* aff. *x‐signatus* (21.8%).

**FIGURE 3:**
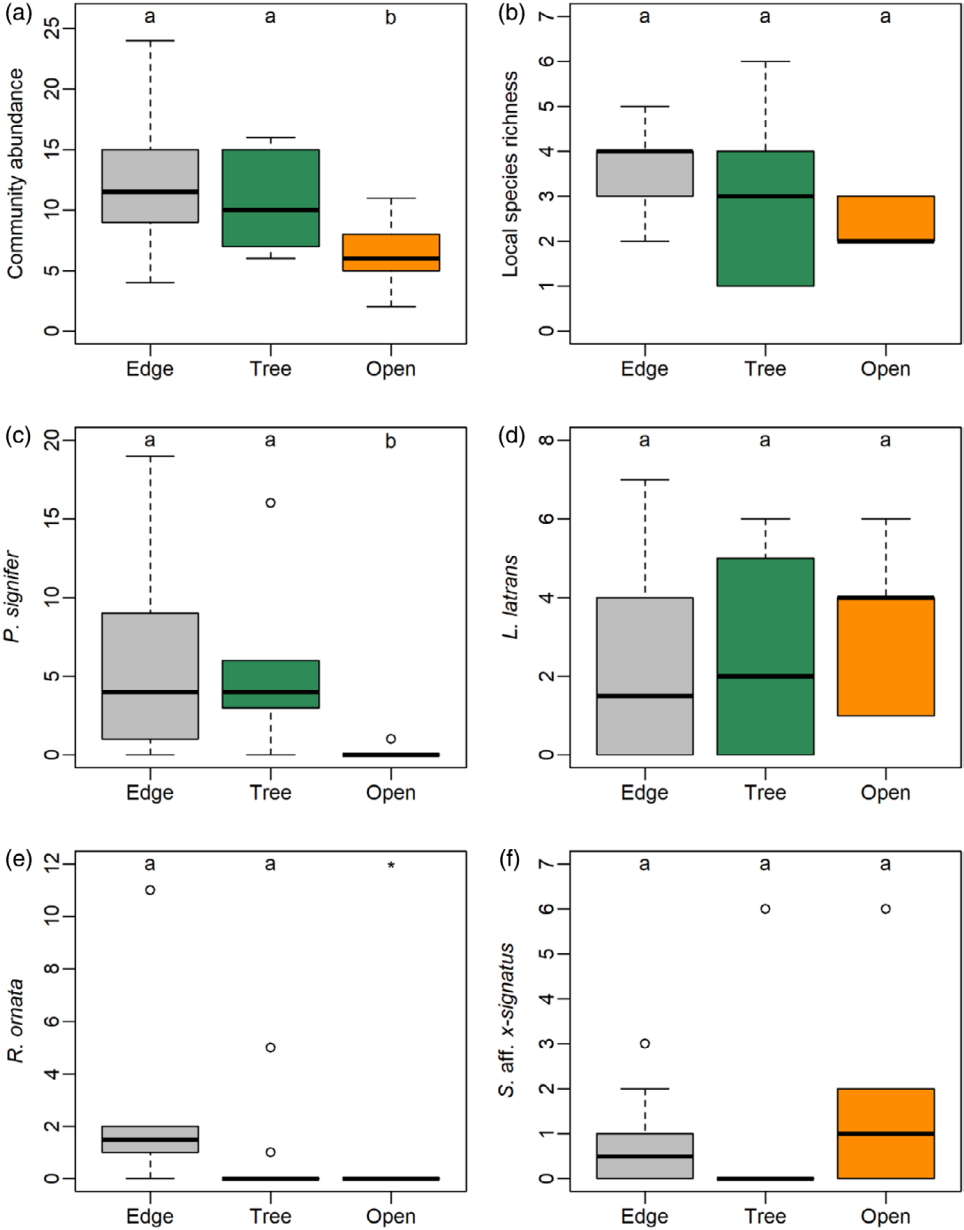
Differences in community abundance and local species richness among experimental treatments. (a) Community abundance; (b) local species richness; (c) abundance of *Physalaemus signifer*; (d) abundance of *Leptodactylus latrans*; (e) abundance of *Rhinella ornata*; (f) abundance of *Scinax* aff. *x‐signatus*. Edge = ponds located near the edge of a continuous forest; Tree = ponds beneath isolated trees; Open = ponds located in open pasture. Each boxplot shows the median (bold horizontal bars), quartiles (boxes), maximum and minimum excluding outliers (bars) and outliers (circles). The different letters above the boxplots indicate statistically significant differences between treatments based on post‐hoc pairwise tests. *Pairwise comparisons involving open ponds were not possible for *R. ornata* because no individuals were recorded in this treatment.

Community abundance differed among treatments (*χ* ^2^ = 13.16, df = 2, *p* = 0.001, *R* ^2^ = 0.49; Figure 3a). Community abundance was significantly lower in open ponds compared to either edge (open‐edge: *z* = 3.54, *p* = 0.001) or tree ponds (open‐tree: *z* = 2.49, *p* = 0.03), but it was similar between edge and tree ponds (edge‐tree: *z* = 1.04, *p* = 0.55; Figure 3a). Local species richness per pond did not differ significantly among treatments (*χ* ^2^ = 2.15, df = 2, *p* = 0.34, *R* ^2^ = 0.17; Figure 3b).

*Physalaemus signifer* was recorded in nine edge ponds (total of 64 individuals), in eight tree ponds (45 individuals) and in only two open ponds (two individuals). *Rhinella ornata* was recorded in eight edge ponds (total of 22 individuals) and in two tree ponds (total of six individuals), with no records in open ponds. On the contrary, *L. latrans* was found in seven edge ponds (total of 21 individuals), in six tree ponds (22 individuals) and in all open ponds (29 individuals), while *S*. aff. *x‐signatus* was found in five edge ponds (total of eight individuals), in one tree pond (six individuals) and in five open ponds (12 individuals). Abundance of *P. signifer* differed significantly among treatments (*χ* ^2^ = 26.05, df = 2, *p* < 0.001, *R* ^2^ = 0.71; Figure 3c), being significantly lower in open ponds compared to either edge or tree ponds (open‐edge: *z* = 4.09, *p* < 0.001; open‐tree: *z* = 3.75, *p* < 0.001), but similar between edge and tree ponds (edge‐tree: *z* = 0.56, *p* = 0.84; Figure 3c). Abundance of *L. latrans* did not differ significantly among treatments (*χ* ^2^ = 1.17, df = 2, *p* = 0.56, *R* ^2^ = 0.06; Figure 3d). Abundance of *R. ornata* differed significantly among treatments (*χ* ^2^ = 15.48, df = 2, *p* < 0.001, *R* ^2^ = 0.61; Figure 3e). The abundance of this species did not differ significantly between edge and tree ponds (*z* = 1.67, *p* = 0.22). Pairwise comparisons involving open ponds were not possible for *R. ornata* because no individuals were recorded in this treatment. Finally, the abundance of *S*. aff. *x‐signatus* did not differ significantly among treatments (*χ* ^2^ = 0.77, df = 2, *p* = 0.68, *R* ^2^ = 0.05; Figure 3f).

### 3.2 Species composition

Considering all ponds from all treatments, the richest family was Hylidae (64.3% of all species), followed by Leptodactylidae (21.4%) and Bufonidae (14.3%). Leptodactylidae was the numerically dominant family in all treatments (tree ponds: 72.0% of the individuals; edge ponds: 68.0%; open ponds: 69.1%), followed by Hylidae (tree ponds: 21.5% of the individuals; edge ponds: 13.6%; open ponds: 30.9%). Bufonidae was only present in tree (6.4% of the individuals) and edge ponds (18.4%).

**FIGURE 4:**
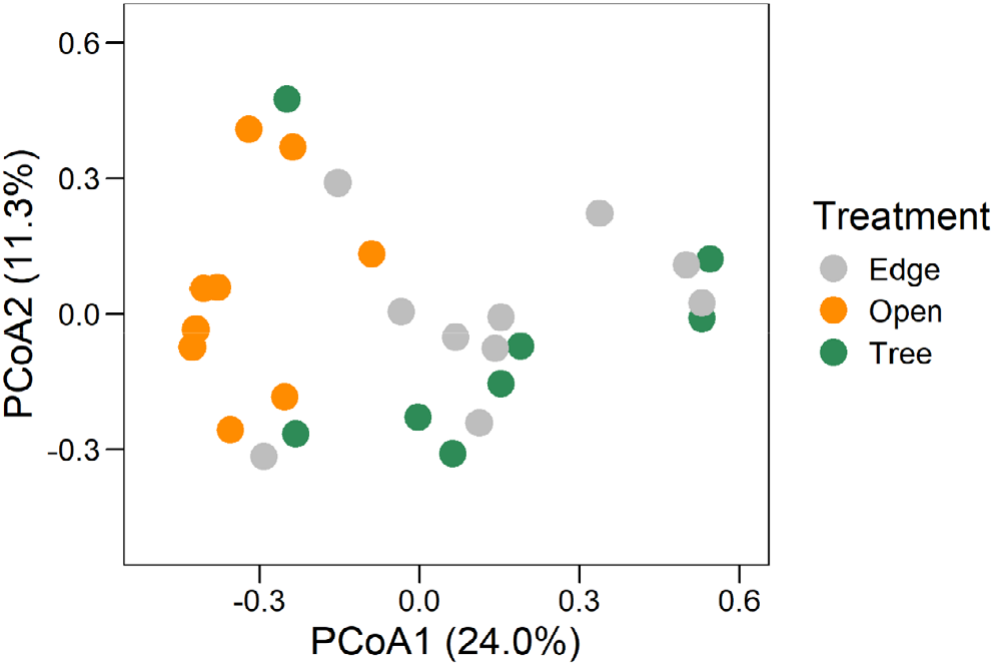
Ordination diagram showing the results of a principal coordinates analysis with dissimilarity among experimental treatments. Edge = ponds located near the edge of a continuous forest; Tree = ponds beneath isolated trees; Open = ponds located in open pasture.

The PCoA indicated that most edge and tree ponds had a similar species composition, whereas open ponds had a markedly different species composition (Figure 4). Indeed, the PERMANOVA tests confirmed that open and tree ponds had a dissimilar community composition (*F*_1,16_ = 5.85, *p* = 0.001, *R* ^2^ = 0.27) as well as open and edge ponds (*F*_1,17_ = 5.80, *p* = 0.001, *R* ^2^ = 0.25). However, tree and edge ponds had a similar community composition (*F*_1,17_ = 0.92; *p* = 0.47, *R* ^2^ = 0.05). The similar community composition of tree and edge ponds, which differed from the composition of open ponds, was probably driven by *P. signifer*, with similar abundance in tree and edge ponds and *R. ornata*, only present in edge and tree ponds (Figure 3c,e).

## 4 DISCUSSION

This study is the first experimental test of the importance of isolated trees for biodiversity. Overall, our results show that isolated trees can play an important role in maintaining frog communities in ponds in fragmented landscapes. Tree ponds had a higher total (landscape‐level) species richness and community abundance than open ponds, corroborating the hypothesis that isolated trees are biodiversity foci (Dunn, 2000; Prevedello et al., 2018). We found that isolated trees may benefit forest‐dependent species and, at the same time, do not reduce the abundance of open‐area species. In addition, local species richness, community abundance and composition of amphibian species were similar in tree and edge ponds, corroborating the hypothesis that isolated trees are keystone structures in landscapes (Fischer et al., 2010; Prevedello et al., 2018). These results reinforce the importance of scattered elements, such as isolated trees, for species distribution and abundance in fragmented landscapes.

### 4.1 Community abundance

The location of ponds in the landscape affected community abundance. Edge and tree ponds had higher community abundance than open ponds. Silva, Oliveira, et al. (2012) also found a higher abundance of frogs in artificial ponds placed at the forest edge than those installed in pasture matrix areas far from forest fragments, reinforcing the importance of tree cover for frog distribution in agricultural landscapes. The continuous forest may act as a source of individuals for edge ponds, which may disperse to nearby ponds. Tree ponds appear to be more attractive to dispersing individuals than open ponds, as the former had higher abundances, despite being at similar distances from the continuous forest. Isolated trees may provide shelter and breeding sites for some species and can be used as stepping stones and short‐term refugia by frogs (Chan‐McLeod & Moy, 2007). Moreover, leaves that fall from these trees can increase frog biomass export by increasing food supply for tadpoles, as many tadpoles consume biofilms and algae, which become more available by the input of plant material and associated nutrients (Earl & Semlitsch, 2012; Schiesari et al., 2009). The higher community abundance in tree ponds compared to open ponds found in our experimental study was also detected in a previous observational study in this same pasture area (Almeida‐Gomes et al., 2016), with ephemeral ponds with isolated trees showing higher local species richness and community abundance than ponds without isolated trees. Taken together, the experimental and observational evidence from our study area corroborates the biodiversity foci hypothesis, which states that scattered trees support higher levels of species richness and/or abundance than nearby open areas (Dunn, 2000; Prevedello et al., 2018).

### 4.2 Species richness

Total (landscape‐level) species richness (i.e. the total number of species found in all ponds of each treatment) was nearly twice as high in tree ponds (11 species) compared to open ponds (six species). As explained above, the presence of isolated trees can provide resources for several species, such as shelters and breeding sites, resulting in a higher total (landscape‐level) species richness. However, we did not detect any statistically significant differences among treatments in terms of local species richness (i.e. number of recorded species per pond) in our experiment (which ranged from one to six species per pond). In a previous observational study in the same area, Almeida‐Gomes et al. (2016) found higher local frog species richness in ponds associated with isolated trees than in ponds without trees. Moreover, local species richness varied from 3 to 14 species per pond in the previous study (Almeida‐Gomes et al., 2016), and pond size was the most important variable explaining variation in local species richness. Many species occurred exclusively in the previous observational study (e.g. *Boana faber*, *Dendropsophus anceps* and *Phyllomedusa burmeisteri*), while one species (*Dendropsophus decipiens*) was only detected in the present study. The main reason for such discrepancy between the observational and the experimental study is probably variation in pond size. While our experimentally created ponds had the same size (nearly 7.5 m^2^), the ephemeral ponds sampled by Almeida‐Gomes et al. (2016) ranged in size from approximately 41 to 820 m^2^. The size of pond habitats can correlate with characteristics that influence colonization‐extinction patterns of anurans, affecting local species richness. For example, ponds with larger areas may have higher habitat heterogeneity (e.g. Almeida‐Gomes et al., 2016) and can receive more individuals by chance through passive sampling (Almeida‐Gomes et al., 2022). Hydroperiod is another pond characteristic that can be correlated with pond size and influence the local species richness of anurans (e.g. Semlitsch et al., 2015) but did not differ among experimental treatments in the present study. Indeed, in the previous observational study in the same area, hydroperiod was the most important variable to explain the composition of frog assemblages in ephemeral ponds (Almeida‐Gomes et al., 2016). Hydroperiod can affect clutch viability, tadpole survival and consequently the species' ability to remain in these habitats (Hartel et al., 2011; Silva, Candeira, & Rossa‐Feres, 2012; Trumbo et al., 2012). Therefore, the absence of significant differences in local species richness among treatments in our study can be explained by the small (and standardized) pond size in our experiment, which limited the number of species in each pond. However, we cannot discard the possibility that differences in local species richness among treatments would emerge if experimental ponds were larger, as suggested by the higher total (landscape‐level) species richness found in tree ponds (*N* = 11) than in open ponds (*N* = 6). Unfortunately, creating 30 larger ponds would be logistically unfeasible in our study area. Nonetheless, our results and those from a previous study in the same area (Almeida‐Gomes et al., 2016) show that even open ponds are useful for frogs in modified habitats, as all open ponds had at least one species and received individuals from either edge (present study; Table S4) or tree ponds (Almeida‐Gomes et al., 2016).

### 4.3 Abundance of the four most abundant species

We found a higher abundance of *P. signifer* and *R. ornata* in edge and tree ponds than in open ponds. This pattern probably reflects the forest habitat of these species, especially *P. signifer* (Almeida‐Gomes et al., 2022). *Physalaemus signifer* is usually found inside forest areas or at their edges, being rarely found in open, matrix areas (Almeida‐Gomes & Rocha, 2014; Haddad et al., 2013). Although *R. ornata* can occur in human‐disturbed areas, it prefers leaf litter in forest areas (Almeida‐Gomes & Rocha, 2014; Maia‐Carneiro et al., 2013). In a previous observational study in the same area with 11 ephemeral ponds in a pasture matrix, these species were found in five (*P. signifer*) and two ponds (*R. ornata*), most of them located near isolated trees (Almeida‐Gomes et al., 2016). The reproductive modes of these species may also explain the differences in abundance we found. *Physalaemus signifer* has two reproductive modes, one with a foam nest floating on ponds and exotrophic tadpoles in ponds (Mode 11) and a foam nest on the humid forest floor and, subsequent to flooding, exotrophic tadpoles in ponds (Mode 28; Haddad et al., 2013). *Rhinella ornata* can have either eggs and exotrophic tadpoles in lentic water (Mode 1) or lotic water (Mode 2; Haddad et al., 2013). Therefore, the occurrence of these species is probably favoured at edge and tree ponds because of the wetter and cooler conditions beneath trees (Manning et al., 2006) and the presence of rivers and streams inside the continuous forest. By creating more favourable conditions, isolated trees may increase population sizes in the landscape and facilitate gene flow among forest remnants, potentially reducing the extinction risk of such forest species.

Conversely, the abundance of species adapted to open areas does not appear to be influenced by the presence of isolated trees or proximity to the forest edge. The abundance of *Leptodactylus latrans* and *Scinax* aff. *x‐signatus* did not differ among treatments. These species were found in a previous study in the same region in continuous forest, forest fragments and matrix areas (Almeida‐Gomes & Rocha, 2014), being considered generalist species (Almeida‐Gomes et al., 2019; Haddad et al., 2013). In the previous observational study (Almeida‐Gomes et al., 2016), *L. latrans* was found in 10 out of 11 ponds and *S*. aff. *x‐signatus* was found in nine out of 11 ponds, including the five ponds with associated trees (Almeida‐Gomes et al., 2016). Therefore, it is not surprising that we did not detect statistical differences among treatments in the abundance of *L. latrans* and *S*. aff. *x‐signatus*, as these species have a generalist habit and are capable of using different habitat types. Furthermore, *L. latrans* had the highest frequency of movements between ponds, suggesting a higher dispersal ability, which could also explain the lack of difference in abundance for this species among treatments. In the previous observational study (Almeida‐Gomes et al., 2016) and in the current one, we observed movements of more than 500 m between ponds by individuals of this species (Table S4). We found other species adapted to disturbed areas in tree ponds, such as species of the genus *Dendropsophus*, suggesting that isolated trees do not have negative impacts on them. Only *Leptodactylus fuscus*, a species adapted to open areas, was found exclusively in open ponds. However, in a previous observational study (Almeida‐Gomes et al., 2016), this species was more abundant in ponds with associated trees. Taken together, our results indicate that isolated trees can benefit forest‐dependent species, without causing negative impacts on species adapted to open environments. However, further studies are necessary to discard the negative impact of isolated trees on species adapted to open environments.

### 4.4 Species composition

The ponds' location in the landscape (treatment) had a direct influence on species composition. The composition was similar between edge and tree ponds, but it was clearly different at open ponds. This result supports the hypothesis that isolated trees are keystone structures in landscapes, as isolated trees can help support communities that are similar to those near forest edges, despite isolated trees occupy a small area in the landscape (Manning et al., 2006; Prevedello et al., 2018). As detailed before, isolated trees can provide higher habitat heterogeneity, a wetter and cooler microclimate and more biomass input inside ponds, compared to vicinal open areas (Almeida‐Gomes et al., 2016; Earl & Semlitsch, 2012; Manning et al., 2006). In fact, the most abundant species in edge and tree ponds was *P. signifer*, a forest‐dependent species that usually does not tolerate open (treeless) areas. Moreover, two other forest‐dependent species, *Pithecopus rohdei* and *R. ornata* (a species with preference for forest habitats), were only found in edge and tree ponds, contributing to the similarity in species composition between these two treatments. In contrast, the most abundant species in open ponds was *L. latrans*. Interestingly, there was a negative and significant correlation (Pearson's *r* = −0.43; *p* = 0.02) between the abundances of the two most abundant species (*P. signifer* and *L. latrans*), which could suggest competition for reproductive sites, as both have the same reproductive strategy in environments outside the forest (foam nest floating on pond and exotrophic tadpoles in ponds—Mode 11; Haddad et al., 2013). Nonetheless, this negative correlation could simply reflect different habitat preferences of these species.

### 4.5 Importance and limitations of using an experimental approach

We controlled in the experimental design several factors that can affect the richness, abundance and composition of amphibian species, such as pond size, hydroperiod, edge slopes, vegetation height, water depth, forest cover and distance to the continuous forest (the latter two only for tree and open ponds). In addition, we also fixed the tree species used in the tree pond treatment (*Ficus* spp.). Therefore, the results we found can be attributed to treatments rather than potentially confounding factors, such as variation in pond size or hydroperiod between tree and open ponds. For this reason, the differences in community abundance, total (landscape‐level) species richness and species composition documented here provide compelling evidence for both the biodiversity foci and the keystone structures hypotheses (Manning et al., 2006; Prevedello et al., 2018).

However, despite the advantages of an experimental approach, we recognise that, as any experiment in ecology, our study may have some limitations, such as the size of the ponds, as discussed earlier, the sampling focused only on adults and the use of a single tree species. We believe that similar results would be observed with a different focal tree species, as trees, regardless of their identity, can provide valuable resources for amphibians. However, further tests are needed to confirm this expectation. Furthermore, we acknowledge that the processes underlying the patterns we found still need to be better understood. For example, the presence of prey, competitors and predators can differ among treatments and affect amphibian communities, as they affect community structure in ephemeral or temporary ponds (Beja & Alcazar, 2003; Richter‐Boix et al., 2007). Finally, sampling communities for more than 1 year could reveal interesting temporal patterns, including how long individuals remain in ponds and seasonal changes in community structure.

## 5 CONCLUSIONS

Our experimental approach provides evidence of the importance of isolated trees in deforested landscapes. This experimental evidence adds to a growing body of observational evidence showing that isolated trees are essential structures in fragmented landscapes worldwide (see reviews in Manning et al., 2006; Prevedello et al., 2018). In our study, isolated trees contributed to increase total (landscape‐level) species richness and community abundance of anuran species in ponds, allowing the maintenance of communities that are similar to those found near the edges of the continuous forest. Despite the growing number of observational studies assessing the importance of isolated trees, and the threats these trees face in human‐modified landscapes, they continue to be mostly neglected in management and conservation plans (Manning et al., 2006; Prevedello et al., 2018). Based on the experimental evidence obtained, we recommend protecting and planting isolated trees (mainly *Ficus* spp.; Cottee‐Jones et al., 2016) in pasture areas, especially near natural ponds. Passive restoration may also contribute to increase the number of isolated trees, which may be promoted by relatively simple actions, such as livestock exclusion to allow tree regeneration (Etchebarne & Brazeiro, 2016). Since several studies have shown that artificial aquatic habitats (e.g. ponds, ditches, drainage channels) can be a valuable conservation strategy for amphibians in disturbed landscapes (e.g. Silva, Oliveira, et al., 2012; Valdez et al., 2021), we believe that the construction of artificial ponds (especially near isolated trees) may have a positive effect on amphibian communities. These low‐cost actions can provide several ecosystem services that might benefit farmers and owners of rural properties, including pollination of crops and shading for cattle in agricultural landscapes (Manning et al., 2006). In addition, these actions can substantially contribute to the long‐term maintenance of anuran and other species in fragmented landscapes, through the increase in landscape connectivity and resilience of ecosystems to further disturbance (Manning et al., 2006).

## AUTHOR CONTRIBUTIONS

Mauricio Almeida‐Gomes and Jayme Augusto Prevedello conceived the idea, defined the sampling design, performed the data analyses and led the writing; Andreza Soares de Siqueira, Vitor Nelson Teixeira Borges‐Júnior, Maria Anita Tozzi and Rodrigo da Fonseca da Silva collected field data; all authors commented on drafts and gave final approval for publication.

## CONFLICT OF INTEREST STATEMENT

We have no conflict of interest to report.

## Supporting information


**Figure S1.** Boxplots comparing the distances of ponds located beneath isolated trees (Tree) or open pasture (Open) to the continuous forest.
**Figure S2.** Boxplots comparing the forest cover surrounding ponds located beneath isolated trees (Tree) or open pasture (Open).
**Figure S3.** Boxplots comparing the hydroperiod of ponds located near the edge of the continuous forest (Edge), beneath isolated trees (Tree) or open pasture (Open).
**Figure S4.** Boxplots comparing vegetation height surrounding ponds located near the edge of the continuous forest (Edge), beneath isolated trees (Tree) or open pasture (Open).
**Figure S5.** Boxplots comparing edge slopes of ponds located near the edge of the continuous forest (Edge), beneath isolated trees (Tree) or open pasture (Open).
**Figure S6.** Boxplots comparing water depth of ponds located near the edge of the continuous forest (Edge), beneath isolated trees (Tree) or open pasture (Open).
**Figure S7.** Predicted differences in amphibian abundance and local species richness among experimental treatments after controlling for hydroperiod and vegetation height.
**Figure S8.** Predicted differences in amphibian abundance and local species richness among experimental treatments after controlling for distance to the continuous forest and forest cover.
**Table S1.** Descriptive variables measured for each experimental pond (*N* = 28).
**Table S2.** Anuran species abundances recorded during Visual Encounter Surveys per treatment, in Cachoeiras de Macacu, Rio de Janeiro, Brazil.
**Table S3.** Anuran species abundances recorded during Visual Encounter Surveys in 28 experimental ponds in Cachoeiras de Macacu, Rio de Janeiro, Brazil.
**Table S4.** Anuran species recaptured during Visual Encounter Surveys in 28 experimental ponds, in Cachoeiras de Macacu, Rio de Janeiro, Brazil.

## Data Availability

Data available from the Dryad Digital Repository https://doi.org/10.5061/dryad.q2bvq83xn (Almeida‐Gomes et al., 2025).
