# Supplementary material for: An experimental test of the importance of isolated trees for frog diversity in tropical landscapes
Source: J Anim Ecol. 2025 Jun 12;94(7):1449–60. doi: 10.1111/1365-2656.70081 (PMC12214440; doi:10.1111/1365-2656.70081)
Supplement: Supplementary file 1 — Figure S1. Boxplots comparing the distances of ponds located beneath isolated trees (Tree) or open pasture (Open) to the continuous forest. Figure S2. Boxplots comparing the forest cover surrounding ponds located beneath isolated trees (Tree) or open pasture (Open). Figure S3. Boxplots comparing the hydroperiod of ponds located near the edge of the continuous forest (Edge), beneath isolated trees (Tree) or open pasture (Open). Figure S4. Boxplots comparing vegetation height surrounding ponds located near the edge of the continuous forest (Edge), beneath isolated trees (Tree) or open pasture (Open). Figure S5. Boxplots comparing edge slopes of ponds located near the edge of the continuous forest (Edge), beneath isolated trees (Tree) or open pasture (Open). Figure S6. Boxplots comparing water depth of ponds located near the edge of the continuous forest (Edge), beneath isolated trees (Tree) or open pasture (Open). Figure S7. Predicted differences in amphibian abundance and local species richness among experimental treatments after controlling for hydroperiod and vegetation height. Figure S8. Predicted differences in amphibian abundance and local species richness among experimental treatments after controlling for distance to the continuous forest and forest cover. Table S1. Descriptive variables measured for each experimental pond (N = 28). Table S2. Anuran species abundances recorded during Visual Encounter Surveys per treatment, in Cachoeiras de Macacu, Rio de Janeiro, Brazil. Table S3. Anuran species abundances recorded during Visual Encounter Surveys in 28 experimental ponds in Cachoeiras de Macacu, Rio de Janeiro, Brazil. Table S4. Anuran species recaptured during Visual Encounter Surveys in 28 experimental ponds, in Cachoeiras de Macacu, Rio de Janeiro, Brazil. [file JANE-94-1449-s001.docx]

**Supporting information**


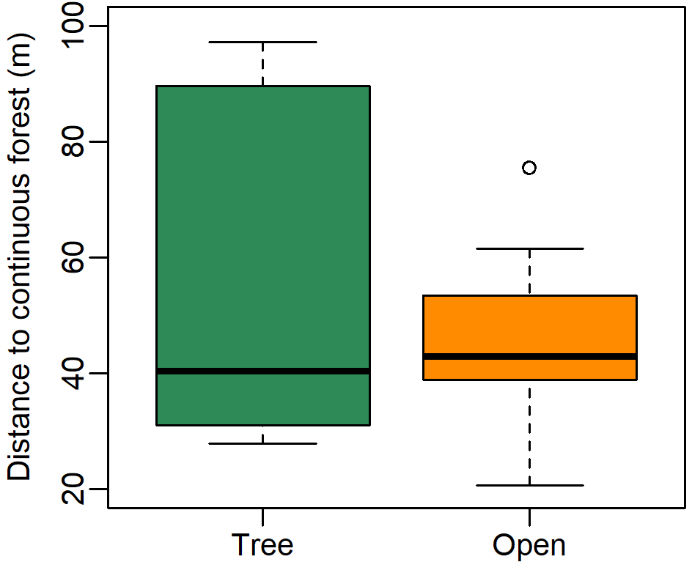


**Figure S1.** Boxplots comparing the distances of ponds located beneath isolated trees (Tree) or open pasture (Open) to the continuous forest. Distances did not differ significantly between the two treatments (F_1,16_ = 0.87, P = 0.37).

**
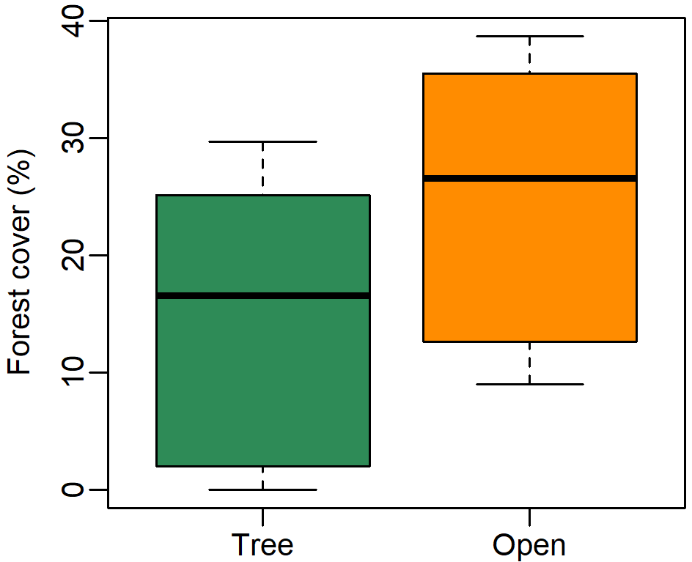
**

**Figure S2.** Boxplots comparing the forest cover surrounding ponds located beneath isolated trees (Tree) or open pasture (Open). Forest cover was estimated within a circular buffer with 100-m radius centered at each pond. Forest cover did not differ significantly between the two treatments (F_1,16_ = 2.49, P = 0.13).

**
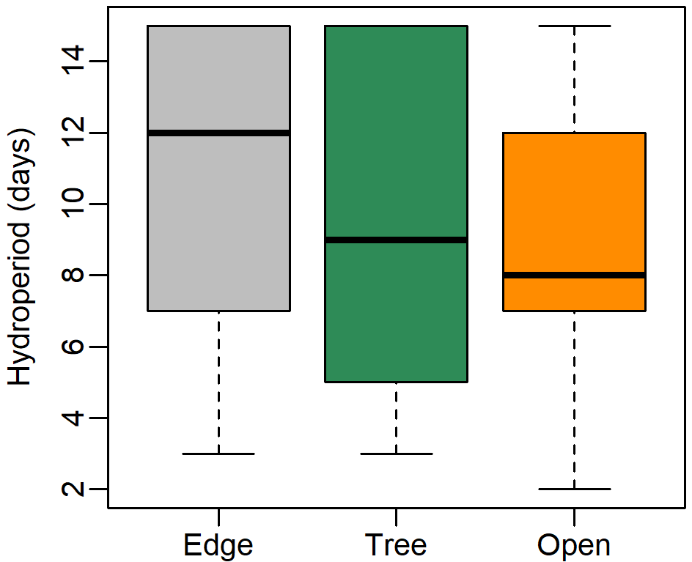
**

**Figure S3.** Boxplots comparing the hydroperiod of ponds located near the edge of the continuous forest (Edge), beneath isolated trees (Tree) or open pasture (Open). Hydroperiod was quantified as the number of sampling days in which the pond had accumulated water. Hydroperiod did not differ significantly among treatments (F_2,25_ = 0.36, P = 0.70).

**
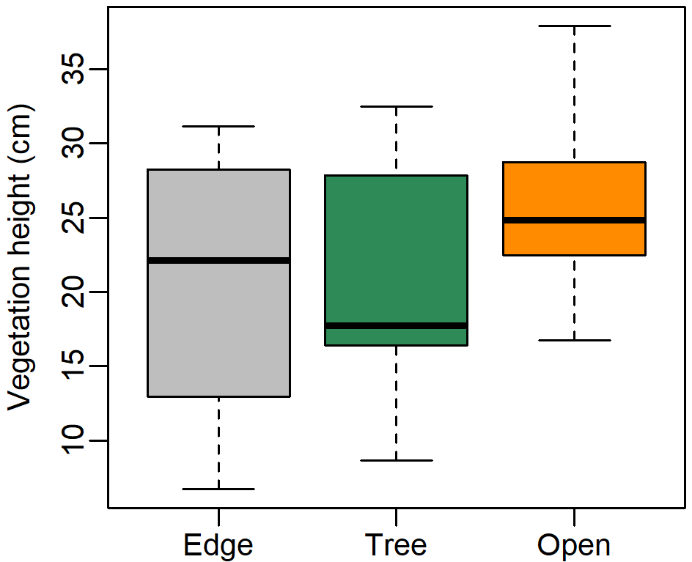
**

**Figure S4.** Boxplots comparing vegetation height surrounding ponds located near the edge of the continuous forest (Edge), beneath isolated trees (Tree) or open pasture (Open). Vegetation height represents the average height of grasses and shrubs measured within 1.5 m of the pond edge at eight points around the pond. Vegetation height did not differ significantly among treatments (F_2,25_ = 1.31, P = 0.29).

**
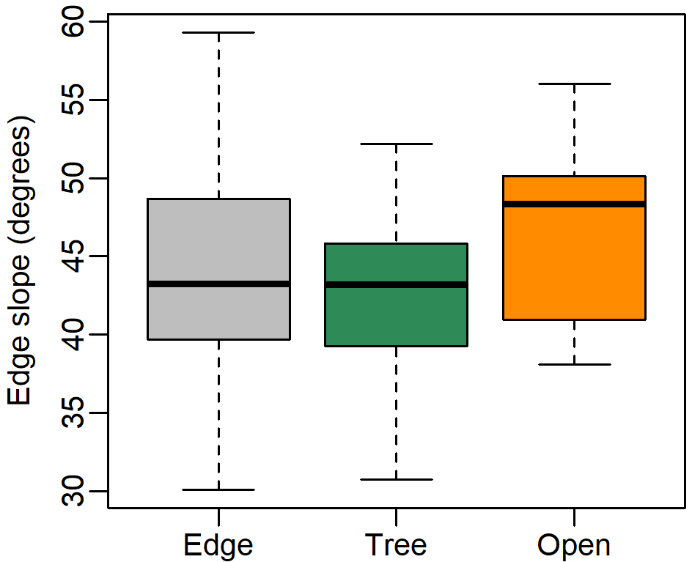
**

**Figure S5.** Boxplots comparing edge slopes of ponds located near the edge of the continuous forest (Edge), beneath isolated trees (Tree) or open pasture (Open). Edge slopes represent the average slope (in degrees) of the two smaller edges of the pond, obtained with a digital clinometer. Edge slope did not differ significantly among treatments (F_2,25_ = 1.00, P = 0.38).

**
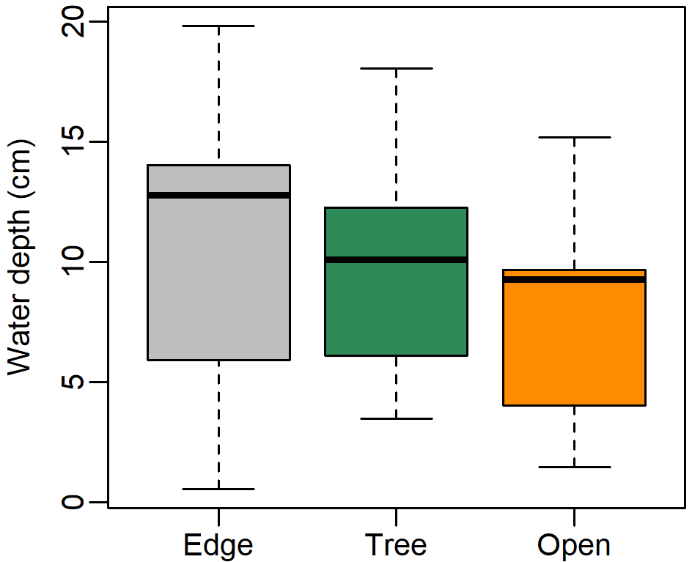
**

**Figure S6.** Boxplots comparing water depth of ponds located near the edge of the continuous forest (Edge), beneath isolated trees (Tree) or open pasture (Open). Water depth was measured at five points spread across each pond, at each of the three sampling days of the five sampling events. For each pond, the five values recorded at the same day were averaged. Then, the average values of each sampling event were averaged. Water depth did not differ significantly among treatments (F_2,25_ = 0.62, P = 0.55).

| **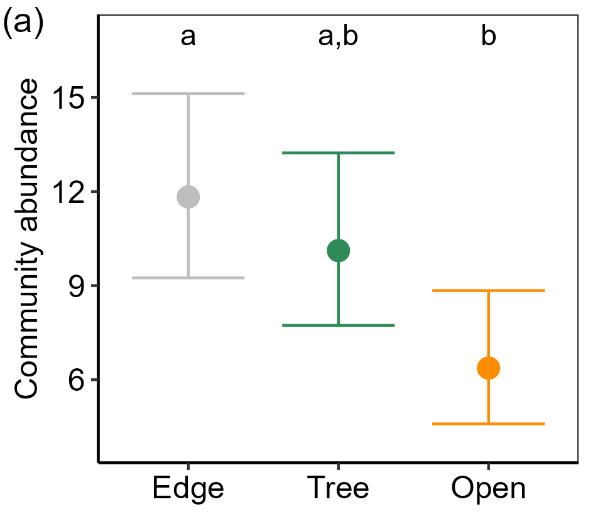** | **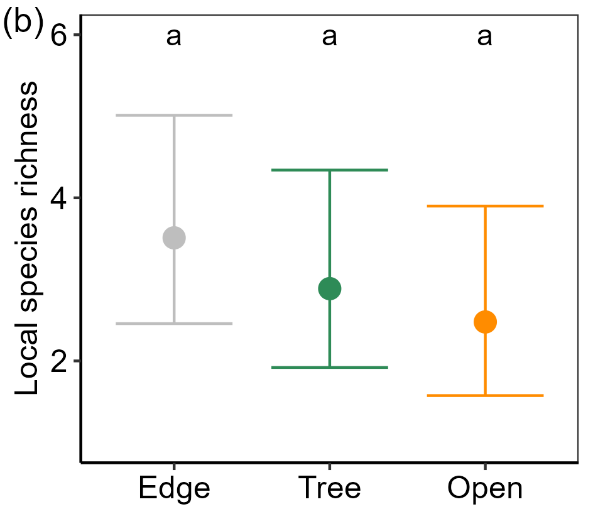** |
| --- | --- |
| **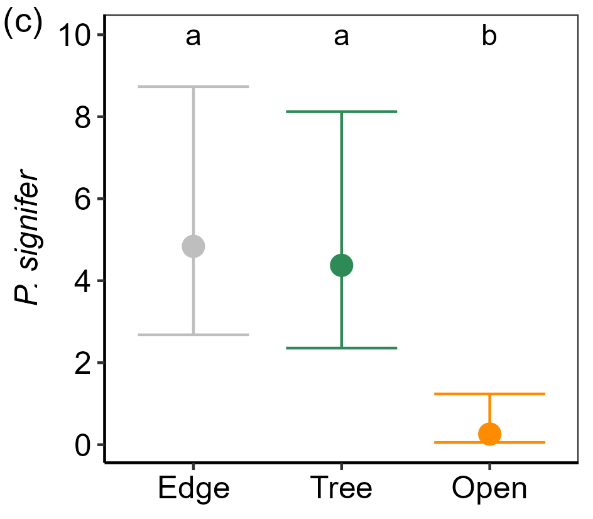** | **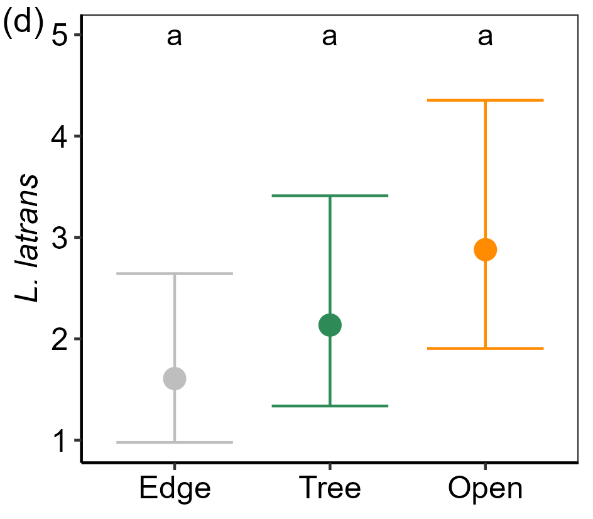** |
| **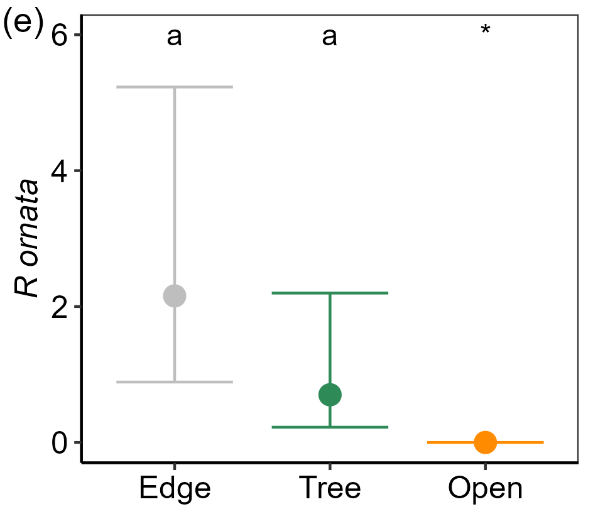** | **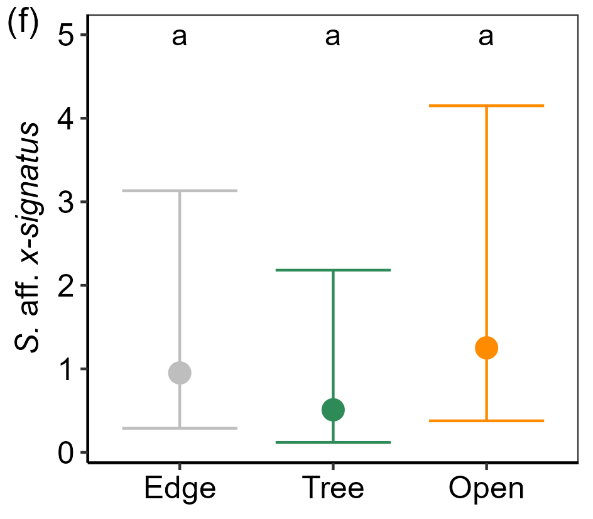** |

**Figure S7.** Predicted differences in amphibian abundance and local species richness among experimental treatments after controlling for hydroperiod and vegetation height. (a) Community abundance; (b) Local species richness; (c) Abundance of *Physalaemus signifer*; (d) Abundance of *Leptodactylus latrans*; (e) Abundance of *Rhinella ornata*; (f) Abundance of *Scinax* aff. *x-signatus*. Edge = ponds located near the edges of the continuous forest; Tree = ponds beneath isolated trees; Open = ponds located in open pasture. Each plot shows the mean and the 95% CI. The different letters above the boxplots indicate statistically significant differences between treatments. *Pairwise comparisons involving open ponds were not possible for *R. ornata* because no individuals were recorded in this treatment. For community abundance (a), the difference between tree and open ponds was marginally significant (P = 0.06).

| **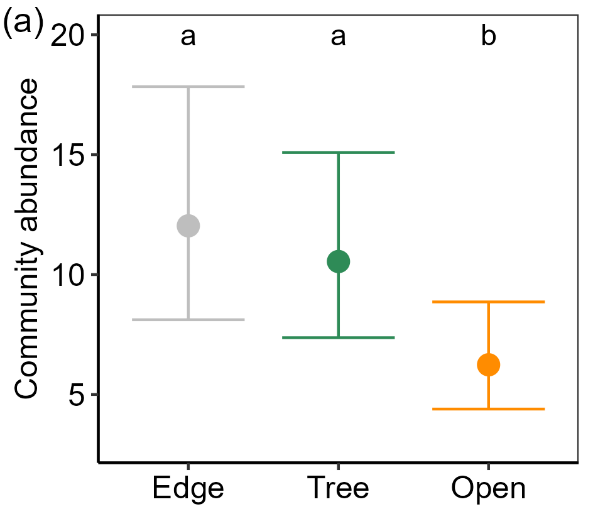** | **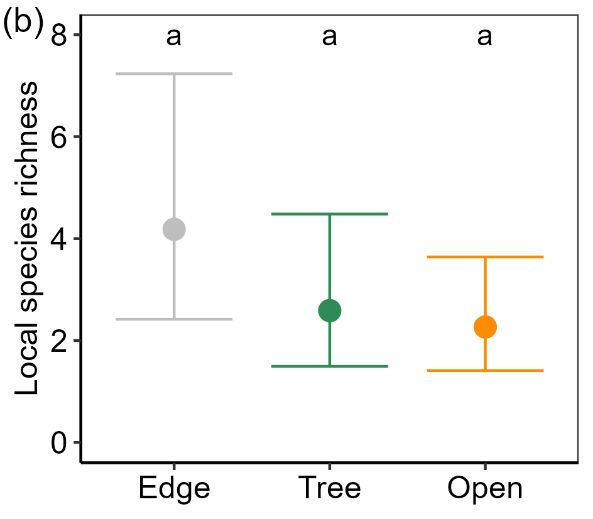** |
| --- | --- |
| **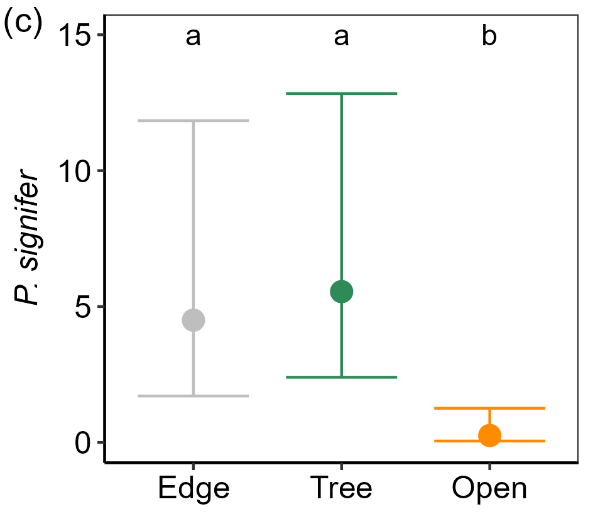** | **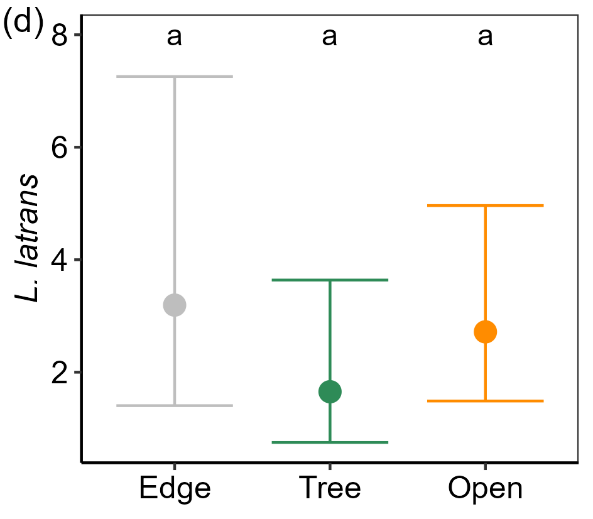** |
| **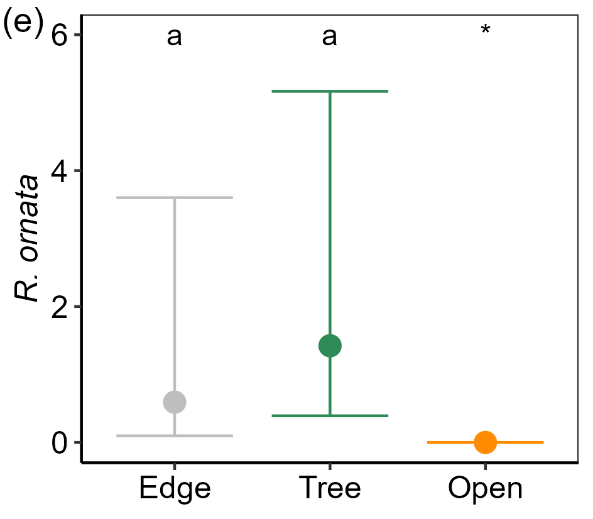** | **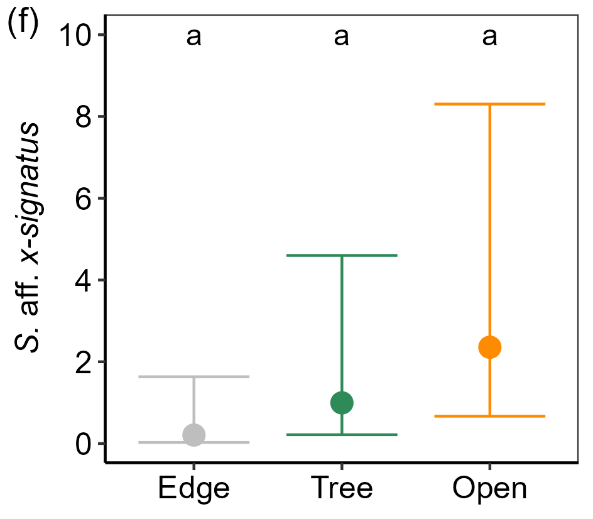** |

**Figure S8.** Predicted differences in amphibian abundance and local species richness among experimental treatments after controlling for distance to the continuous forest and forest cover. (a) Community abundance; (b) Local species richness; (c) Abundance of *Physalaemus signifer*; (d) Abundance of *Leptodactylus latrans*; (e) Abundance of *Rhinella ornata*; (f) Abundance of *Scinax* aff. *x-signatus*. Edge = ponds located near the edges of a continuous forest; Tree = ponds beneath isolated trees; Open = ponds located in open pasture. Each plot shows the mean and the 95% CI. The different letters above the boxplots indicate statistically significant differences between treatments. * Pairwise comparisons involving open ponds were not possible for *R. ornata* because no individuals were recorded in this treatment.

**Table S1.** Descriptive variables measured for each experimental pond (N = 28). Two experimental ponds (one in open pasture and one beneath an isolated tree) did not accumulate water and were excluded from the analyses. Forest cover was measured at a circular buffer of 100-m radius. Distance = distance to the edge of the continuous forest.

|  | Treatment | Forest cover (%) | Distance (m) | Hydroperiod (days) | Vegetation height (cm) | Edge slope (degrees) | Water depth (cm) |
| --- | --- | --- | --- | --- | --- | --- | --- |
| P1 | Open | 36.3 | 43.0 | 6 | 19.7 | 39.5 | 2.8 |
| P2 | Tree | 12.5 | 65.8 | 15 | 31.4 | 32.1 | 12.3 |
| P3 | Tree | 2.0 | 89.6 | 5 | 27.8 | 30.7 | 6.1 |
| P5 | Open | 12.7 | 53.5 | 8 | 28.7 | 56.0 | 9.7 |
| P6 | Open | 26.6 | 28.9 | 7 | 22.5 | 50.1 | 4.0 |
| P7 | Open | 38.7 | 20.6 | 2 | 27.9 | 38.1 | 1.5 |
| P8 | Open | 9.0 | 61.5 | 12 | 16.7 | 48.3 | 5.2 |
| P9 | Tree | 29.7 | 27.9 | 14 | 17.7 | 45.8 | 4.9 |
| P10 | Tree | 0.0 | 96.2 | 9 | 16.4 | 43.2 | 10.5 |
| P11 | Edge | 31.9 | 0.5 | 7 | 6.7 | 55.7 | 5.3 |
| P12 | Edge | 48.4 | 0.5 | 12 | 10.4 | 48.6 | 7.2 |
| P13 | Tree | 16.5 | 31.0 | 9 | 23.0 | 41.6 | 9.1 |
| P14 | Tree | 25.2 | 29.1 | 5 | 8.6 | 39.2 | 15.9 |
| P15 | Open | 32.6 | 40.4 | 15 | 24.8 | 48.8 | 9.3 |
| P16 | Edge | 42.3 | 0.5 | 12 | 22.3 | 39.7 | 13.1 |
| P17 | Open | 12.6 | 52.4 | 8 | 37.9 | 48.2 | 15.2 |
| P18 | Edge | 54.8 | 0.5 | 15 | 21.9 | 44.8 | 12.5 |
| P19 | Edge | 47.4 | 0.5 | 15 | 29.4 | 30.1 | 14.3 |
| P20 | Edge | 35.2 | 0.5 | 5 | 22.2 | 41.6 | 0.5 |
| P21 | Tree | 28.1 | 34.2 | 3 | 32.5 | 49.3 | 3.5 |
| P22 | Open | 9.7 | 75.5 | 11 | 29.1 | 40.9 | 9.4 |
| P23 | Tree | 17.5 | 40.4 | 15 | 17.7 | 52.2 | 18.1 |
| P24 | Tree | 1.1 | 97.1 | 15 | 15.5 | 44.3 | 10.1 |
| P25 | Open | 35.5 | 38.9 | 15 | 23.8 | 52.6 | 15.2 |
| P26 | Edge | 57.1 | 0.5 | 15 | 18.6 | 45.9 | 13.0 |
| P27 | Edge | 61.6 | 0.5 | 3 | 28.2 | 41.6 | 5.9 |
| P28 | Edge | 46.9 | 0.5 | 12 | 12.9 | 59.3 | 14.0 |
| P30 | Edge | 31.6 | 0.5 | 15 | 31.2 | 38.9 | 19.8 |

**Table S2.** Anuran species abundances recorded during Visual Encounter Surveys per treatment, in Cachoeiras de Macacu, Rio de Janeiro, Brazil. Ten ponds were located near the edge of the continuous forest (Edge ponds), nine beneath isolated trees (Tree ponds) and nine in open pasture (Open ponds). Two experimental ponds (one in open pasture and one beneath an isolated tree) did not accumulate water and were excluded from the analyses. The last two columns show the presence (0) or absence (1) of each species in the dry (D) and the wet (W) season separately.

|  | **Edge ponds** | **Tree ponds** | **Open ponds** | **D** | **W** |
| --- | --- | --- | --- | --- | --- |
| **Bufonidae** |  |  |  |  |  |
| *Rhinella icterica* | 1 | 0 | 0 | 0 | 1 |
| *Rhinella ornata* | 22 | 6 | 0 | 1 | 1 |
| **Hylidae** |  |  |  |  |  |
| *Boana albomarginata* | 4 | 4 | 0 | 0 | 1 |
| *Boana semilineata* | 1 | 0 | 1 | 1 | 1 |
| *Dendropsophus decipiens* | 0 | 2 | 0 | 0 | 1 |
| *Dendropsophus elegans* | 0 | 1 | 0 | 0 | 1 |
| *Dendropsophus meridianus* | 2 | 1 | 0 | 0 | 1 |
| *Dendropsophus minutus* | 0 | 2 | 0 | 0 | 1 |
| *Dendropsophus seniculus* | 1 | 2 | 4 | 1 | 1 |
| *Pithecopus rohdei* | 1 | 2 | 0 | 0 | 1 |
| *Scinax* aff. *x-signatus* | 8 | 6 | 12 | 1 | 1 |
| **Leptodactylidae** |  |  |  | 0 |  |
| *Leptodactylus fuscus* | 0 | 0 | 7 | 0 | 1 |
| *Leptodactylus latrans* | 21 | 22 | 29 | 1 | 1 |
| *Physalaemus signifer* | 64 | 45 | 2 | 1 | 1 |
| **TOTAL ABUNDANCE** | **125** | **93** | **55** |  |  |

**Table S3.** Anuran species abundances recorded during Visual Encounter Surveys in 28 experimental ponds in Cachoeiras de Macacu, Rio de Janeiro, Brazil. Two experimental ponds (one in open pasture and one beneath an isolated tree) did not accumulate water and were excluded from the analyses. Bo_al = *Boana albomarginata*; Bo_se = *Boana semilineata*; De_de = *Dendropsophus decipiens*; De_el = *Dendropsophus elegans*; De_me = *Dendropsophus meridianus*; De_mi = *Dendropsophus minutus*; De_se = *Dendropsophus seniculus*; Le_fu = *Leptodactylus fuscus*; Le_la = *Leptodactylus latrans*; Ph_si = *Physalaemus signifer*; Pi_ro = *Pithecopus rohdei*; Rh_ic = *Rhinella icterica*; Rh_or = *Rhinella ornata*; Sc_xs = *Scinax* aff. *x-signatus*.

|  | Treatment | Bo_al | Bo_se | De_de | De_el | De_me | De_mi | De_se | Le_fu | Le_la | Ph_si | Pi_ro | Rh_ic | Rh_or | Sc_xs |
| --- | --- | --- | --- | --- | --- | --- | --- | --- | --- | --- | --- | --- | --- | --- | --- |
| P1 | Open | 0 | 1 | 0 | 0 | 0 | 0 | 0 | 0 | 4 | 1 | 0 | 0 | 0 | 0 |
| P2 | Tree | 3 | 0 | 0 | 0 | 0 | 0 | 1 | 0 | 5 | 3 | 2 | 0 | 1 | 0 |
| P3 | Tree | 0 | 0 | 0 | 0 | 0 | 0 | 0 | 0 | 0 | 6 | 0 | 0 | 0 | 0 |
| P5 | Open | 0 | 0 | 0 | 0 | 0 | 0 | 0 | 0 | 1 | 0 | 0 | 0 | 0 | 1 |
| P6 | Open | 0 | 0 | 0 | 0 | 0 | 0 | 0 | 3 | 1 | 1 | 0 | 0 | 0 | 0 |
| P7 | Open | 0 | 0 | 0 | 0 | 0 | 0 | 2 | 0 | 1 | 0 | 0 | 0 | 0 | 0 |
| P8 | Open | 0 | 0 | 0 | 0 | 0 | 0 | 2 | 0 | 4 | 0 | 0 | 0 | 0 | 2 |
| P9 | Tree | 0 | 0 | 0 | 0 | 0 | 0 | 0 | 0 | 0 | 16 | 0 | 0 | 0 | 0 |
| P10 | Tree | 0 | 0 | 2 | 1 | 1 | 0 | 0 | 0 | 2 | 4 | 0 | 0 | 0 | 0 |
| P11 | Edge | 0 | 0 | 0 | 0 | 0 | 0 | 0 | 0 | 1 | 19 | 0 | 0 | 1 | 3 |
| P12 | Edge | 0 | 0 | 0 | 0 | 0 | 0 | 0 | 0 | 4 | 5 | 0 | 0 | 1 | 0 |
| P13 | Tree | 0 | 0 | 0 | 0 | 0 | 0 | 0 | 0 | 3 | 5 | 0 | 0 | 0 | 0 |
| P14 | Tree | 0 | 0 | 0 | 0 | 0 | 0 | 0 | 0 | 0 | 6 | 0 | 0 | 0 | 0 |
| P15 | Open | 0 | 0 | 0 | 0 | 0 | 0 | 0 | 1 | 4 | 0 | 0 | 0 | 0 | 6 |
| P16 | Edge | 0 | 0 | 0 | 0 | 0 | 0 | 1 | 0 | 0 | 16 | 0 | 0 | 2 | 0 |
| P17 | Open | 0 | 0 | 0 | 0 | 0 | 0 | 0 | 0 | 4 | 0 | 0 | 0 | 0 | 1 |
| P18 | Edge | 3 | 0 | 0 | 0 | 0 | 0 | 0 | 0 | 2 | 3 | 0 | 0 | 2 | 1 |
| P19 | Edge | 0 | 0 | 0 | 0 | 0 | 0 | 0 | 0 | 4 | 8 | 0 | 0 | 1 | 2 |
| P20 | Edge | 0 | 1 | 0 | 0 | 0 | 0 | 0 | 0 | 1 | 1 | 0 | 0 | 0 | 1 |
| P21 | Tree | 0 | 0 | 0 | 0 | 0 | 2 | 1 | 0 | 1 | 0 | 0 | 0 | 0 | 6 |
| P22 | Open | 0 | 0 | 0 | 0 | 0 | 0 | 0 | 3 | 6 | 0 | 0 | 0 | 0 | 0 |
| P23 | Tree | 0 | 0 | 0 | 0 | 0 | 0 | 0 | 0 | 6 | 4 | 0 | 0 | 5 | 0 |
| P24 | Tree | 1 | 0 | 0 | 0 | 0 | 0 | 0 | 0 | 5 | 1 | 0 | 0 | 0 | 0 |
| P25 | Open | 0 | 0 | 0 | 0 | 0 | 0 | 0 | 0 | 4 | 0 | 0 | 0 | 0 | 2 |
| P26 | Edge | 0 | 0 | 0 | 0 | 0 | 0 | 0 | 0 | 2 | 2 | 0 | 1 | 2 | 1 |
| P27 | Edge | 0 | 0 | 0 | 0 | 0 | 0 | 0 | 0 | 0 | 1 | 0 | 0 | 11 | 0 |
| P28 | Edge | 1 | 0 | 0 | 0 | 2 | 0 | 0 | 0 | 0 | 9 | 1 | 0 | 0 | 0 |
| P30 | Edge | 0 | 0 | 0 | 0 | 0 | 0 | 0 | 0 | 7 | 0 | 0 | 0 | 2 | 0 |

**Table S4.** Anuran species recaptured during Visual Encounter Surveys in 28 experimental ponds, in Cachoeiras de Macacu, Rio de Janeiro, Brazil. The arrows indicate the direction of the recorded movement.

|  | **Capture** | **Recapture** | **Distance (m)** | **Treatment** |
| --- | --- | --- | --- | --- |
| **Bufonidae** |  |  |  |  |
| *Rhinella ornata* | P18 | P18 | 0 | Edge |
| *Rhinella ornata* | P18 | P18 | 0 | Edge |
| *Rhinella ornata* | P23 | P23 | 0 | Tree |
| *Rhinella ornata* | P27 | P27 | 0 | Edge |
| **Hylidae** |  |  |  |  |
| *Boana albomarginata* | P24 | P24 | 0 | Tree |
| *Boana semilineata* | P20 | P20 | 0 | Edge |
| *Dendropsophus meridianus* | P28 | P28 | 0 | Edge |
| *Dendropsophus minutus* | P21 | P21 | 0 | Tree |
| **Leptodactylidae** |  |  |  |  |
| *Leptodactylus fuscus* | P6 | P6 | 0 | Open |
| *Leptodactylus latrans* | P30 | P30 | 0 | Edge |
| *Leptodactylus latrans* | P30 | P30 | 0 | Edge |
| *Leptodactylus latrans* | P30 | P24 | 131.6 | Edge→Tree |
| *Leptodactylus latrans* | P22 | P22 | 0 | Open |
| *Leptodactylus latrans* | P19 | P19 | 0 | Edge |
| *Leptodactylus latrans* | P15 | P15 | 0 | Open |
| *Leptodactylus latrans* | P12 | P12 | 0 | Edge |
| *Leptodactylus latrans* | P12 | P12 | 0 | Edge |
| *Leptodactylus latrans* | P24 | P24 | 0 | Tree |
| *Leptodactylus latrans* | P12 | P12 | 0 | Edge |
| *Leptodactylus latrans* | P1 | P1 | 0 | Open |
| *Leptodactylus latrans* | P18 | P25 | 505.5 | Edge→Open |
| *Leptodactylus latrans* | P13 | P13 | 0 | Tree |
| *Leptodactylus latrans* | P26 | P26 | 0 | Edge |
| *Physalaemus signifer* | P9 | P9 | 0 | Tree |
| *Physalaemus signifer* | P9 | P9 | 0 | Tree |
| *Physalaemus signifer* | P9 | P9 | 0 | Tree |
| *Physalaemus signifer* | P9 | P9 | 0 | Tree |
| *Physalaemus signifer* | P9 | P9 | 0 | Tree |
| *Physalaemus signifer* | P9 | P9 | 0 | Tree |
| *Physalaemus signifer* | P10 | P16 | 163.9 | Tree→Edge |
| *Physalaemus signifer* | P16 | P16 | 0 | Edge |
| *Physalaemus signifer* | P28 | P28 | 0 | Edge |
| *Physalaemus signifer* | P16 | P16 | 0 | Edge |
| *Physalaemus signifer* | P13 | P13 | 0 | Tree |
| *Physalaemus signifer* | P13 | P13 | 0 | Tree |
| *Physalaemus signifer* | P12 | P12 | 0 | Edge |
| *Physalaemus signifer* | P11 | P11 | 0 | Edge |
| *Physalaemus signifer* | P3 | P3 | 0 | Tree |
